# Unfixed Endogenous Retroviral Insertions in the Human Population

**DOI:** 10.1128/JVI.00919-14

**Published:** 2014-09

**Authors:** Emanuele Marchi, Alex Kanapin, Gkikas Magiorkinis, Robert Belshaw

**Affiliations:** a Department of Zoology, University of Oxford, Oxford, United Kingdom; b The Wellcome Trust Centre for Human Genetics, Oxford, United Kingdom; c Virus Reference Department, Public Health England, London, United Kingdom; d School of Biomedical and Healthcare Sciences, Plymouth University, Plymouth, United Kingdom

## Abstract

One lineage of human endogenous retroviruses (HERVs), HERV-K(HML2), is upregulated in many cancers, some autoimmune/inflammatory diseases, and HIV-infected cells. Despite 3 decades of research, it is not known if these viruses play a causal role in disease, and there has been recent interest in whether they can be used as immunotherapy targets. Resolution of both these questions will be helped by an ability to distinguish between the effects of different integrated copies of the virus (loci). Research so far has concentrated on the 20 or so recently integrated loci that, with one exception, are in the human reference genome sequence. However, this viral lineage has been copying in the human population within the last million years, so some loci will inevitably be present in the human population but absent from the reference sequence. We therefore performed the first detailed search for such loci by mining whole-genome sequences generated by next-generation sequencing. We found a total of 17 loci, and the frequency of their presence ranged from only 2 of the 358 individuals examined to over 95% of them. On average, each individual had six loci that are not in the human reference genome sequence. Comparing the number of loci that we found to an expectation derived from a neutral population genetic model suggests that the lineage was copying until at least ∼250,000 years ago.

**IMPORTANCE** About 5% of the human genome sequence is composed of the remains of retroviruses that over millions of years have integrated into the chromosomes of egg and/or sperm precursor cells. There are indications that protein expression of these viruses is higher in some diseases, and we need to know (i) whether these viruses have a role in causing disease and (ii) whether they can be used as immunotherapy targets in some of them. Answering both questions requires a better understanding of how individuals differ in the viruses that they carry. We carried out the first careful search for new viruses in some of the many human genome sequences that are now available thanks to advances in sequencing technology. We also compared the number that we found to a theoretical expectation to see if it is likely that these viruses are still replicating in the human population today.

## INTRODUCTION

Endogenous retroviruses (ERVs) are retroviruses that have integrated into germ line cells and become inherited in a Mendelian fashion (1). The human genome has ∼100,000 ERV loci resulting from proliferations of ∼50 independent invasions of the genome from free-living (exogenous) retroviruses (2, 3). Only one ERV lineage has continued to replicate in the human population within the last few million years. This lineage is HERV-K(HML2), which for brevity we call HK2. There are ∼1,000 HK2 loci in the human reference genome, and these have integrated over the last ∼35 million years. During the repeated rounds of host replication in this period, most full-length integrated ERV loci (proviruses) have been converted to the relict, non-protein-coding structure known as a solo long terminal repeat (LTR) by recombination, and all of the remainder have acquired premature stop codons and/or indels that cause frameshifts. All loci in the reference genome are therefore replication defective, and only 24 loci retain full-length open reading frames (ORFs) in at least one of their genes (4). RNA transcription and protein expression of HK2 and other ERVs are elevated in many cancers, some autoimmune/inflammatory diseases, and HIV infection, and there has been a long and unresolved search for a causal role in disease (5–7). More recently, this elevation of protein expression in disease has led to research into their potential as immunotherapy targets for cancer and HIV treatment (8–12).

To determine the possible role of HK2 in both pathogenesis and therapy, we need to distinguish between the different loci in the human population (6, 24). RNA transcription levels vary between loci (15), and all known cases of ERVs or elements related to ERVs involved in human disease or therapy have been related to individual loci (16–18). Some loci are in all humans, but these loci are by definition old (because they have had time to drift to fixation), tend to be more degraded, and hence, are less likely to be pathogenic or to be capable of producing proteins in cancerous or HIV-infected cells. In contrast, loci present in only some individuals (unfixed loci, where some individuals carry only the preintegration site) are, on average, younger and hence more likely to produce proteins and perhaps even be capable of replication (19). Some diseases might therefore be associated only with specific unfixed loci, and the efficacy and safety of any HK2-based immunotherapy might vary between individuals because of differences in their complement of unfixed loci. Until now, research has been based on our knowledge of loci that are in the human reference genome plus the one full-length locus that is known to be in the human population but is not in the reference, K113 (20).

Next-generation sequencing (NGS) allows us now to examine almost complete genomes of many individuals, and here we report the first thorough mining by NGS of whole genome sequences for HK2 loci that are not in the human genome reference sequence. A recent study investigating the copying of transposable elements in cancer genomes reported finding 31 such HK2 loci (21), but not all of these were examined in detail. We designed a new method that uses two approaches to the initial detection of previously unknown loci and a final validation step that allows visual confirmation of the integration by aligning the NGS reads. We used this approach on the genomes of cancer patients for several reasons: (i) cancer tissue genomes are typically more deeply sequenced than those in healthy tissues, (ii) there is a putative causal link between HK2 and cancer (22), and (iii) other transposable elements are known to be mobilized in cancer cells (23). Once the loci were identified, we then measured their frequency in a much larger number of patients with a range of diseases.

Another unresolved question is whether HK2 is increasing its copy number within the germ line of the human population today (19, 24). No locus capable of replication has been found, but that may merely reflect the relatively small number of individuals examined. We therefore compared the number of loci found to an expected number that we derived from a neutral population genetic model that assumes a constant rate of replication from our common ancestor with the chimpanzee until today.

## MATERIALS AND METHODS

### The data.

We analyzed two whole-genome-sequence data sets. The first one consisted of data from 26 Cancer Genome Atlas Project (TCGA) patients. Paired cancer and germ line genomes (sequenced from blood or healthy solid tissue) at a 39 times average coverage were available for each patient. All sequences were obtained with the Illumina paired-end technology, which gave 100-nucleotide (nt) reads, and downloaded as BAM files from the University of California, Santa Cruz (UCSC), Cancer Genomics Hub (CGhub). The 26 patients included 10 with breast cancer, 6 with ovarian cancer, 5 with squamous cell lung cancer, and 5 with glioblastoma multiforme brain cancers. Analyses were done on a six-core (12-processing-unit) computer with 64 Gb of random-access memory and 12 Tb of storage.

The second data set consisted of data from 332 patients participating in the WGS500 project. The WGS500 project is a collaboration between the University of Oxford and Illumina and contains several disease cohorts, each of which is focused on particular rare Mendelian diseases and various cancers. We used 410 whole-genome sequences from a total of 332 patients sequenced by 100-nt paired-end protocols on either an Illumina HiSeq 2000 instrument or an Illumina HiSeq 2500 instrument in standard mode, with a mixture of v2.5 and v3.0 chemistries, to at least 25 times average coverage. The ethnic origin of the patients is mostly Caucasian and Asian. The data set includes several family trios and quartets, but we excluded data from children from our analyses. The computation procedures have been run on the Wellcome Trust Centre for Human Genetics high-performance computing cluster containing ∼1,000 cores and 1.5 Pb of storage.

### Mining pipeline.

We first searched the 26 TCGA patient genomes manually for loci not in the reference genome and then measured their frequency in the 332 WGS500 patients. Programs such as RetroSeq (25) (run in the discovery phase) detect many candidate loci, most of which turn out on closer inspection not to be integrations but result from some other polymorphisms, e.g., a group of transposable elements called SINE/variable-number tandem-repeat/Alu (SVA) elements that contain fragments of an HK2 LTR (26) or differences in the degree of fragmentation of a fixed HK2 locus (see Methods and Fig. S1 and S2 in the supplemental material). Also, mining of whole-genome sequences for transposable elements is relatively easy within the single-copy DNA regions of the genome but becomes difficult in repeat regions, where reads can often no longer be mapped incontrovertibly to one location only. Due to the high number of repeats, 63% of the human genome sequence is either a repeat or is single-copy DNA that is within 100 nt of a repeat. We therefore developed a new approach that combined the use of paired-end reads by RetroSeq with (i) the detection of chimeric reads that span the integration and (ii) a final visual inspection.

In TCGA patients, we also searched for evidence of somatic integrations, which would manifest themselves as loci in cancer genomes that were not also present in the corresponding germ line genome. Careful inspection, however, was needed because we would expect such loci to be present in only a small minority of cells and, hence, NGS reads, depending upon at which division in tumor growth the integration occurred.

We follow the nomenclatural system used by Subramanian et al. (4) for full-length loci (proviruses) in the human reference genome. The taxonomic problems of ERV groups and loci are discussed by Mayer et al. (3).

### (i) Pipeline for TCGA genomes.

**FIG 1:**
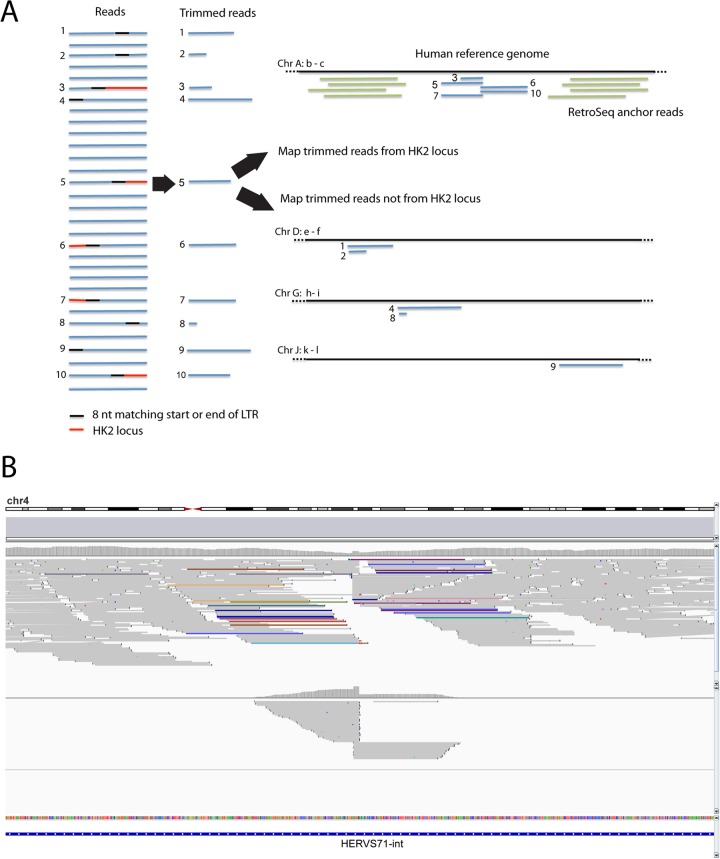
Detection of integrations not in the human reference sequence. (A) Schematic of pipeline for finding loci showing how mapping of trimmed reads is linked to result of RetroSeq analysis. Mapping creates a cluster of trimmed reads that are derived from HK2 loci, which are inside the cluster of RetroSeq anchor reads. In contrast, trimmed reads derived from other regions by chance sequence similarity are scattered around the genome. The next stage is confirmation of integration by BreakAlign analysis. Chr, chromosome. (B) Example of the Integrative Genomics Viewer genome browser (49) screenshot showing evidence for the 4q22.3 locus (from chromosome 4 [chr4], coordinates 9602941 to 9603548). (Top) Mapping of all reads with colored ones representing RetroSeq anchors (see Materials and Methods; the color shows the chromosome on which the mate has been mapped to another HK2 locus in the reference genome); (middle) mapping of trimmed reads, with the coverage at each nucleotide position being shown above the reads. The short overlap representing the 6-nt target site duplication causes a doubling of coverage at these 6 nt, forming the tower in the characteristic submarine-shaped profile of the coverage. (Bottom) RepeatMasker track. In this instance, the HK2 virus has integrated into an existing ERV belonging to another lineage, HERVS71.

There are three steps in the pipeline for TCGA genomes. The first two are summarized in Fig. 1.

In the first step, the paired reads are run through RetroSeq, which returns a list of mapped reads (henceforth called the anchor) whose other end (henceforth called the mate) both (i) did not match the adjacent region of the reference human genome sequence and (ii) matched a reference transposable element. As a reference, we downloaded from GenBank (accession number AY037928) the LTR of K113, which is the most intact locus known (20). The LTR is the 300- to 1,000-nt region at the beginning and end of an ERV integration (almost 1,000 nt long in HK2), and the two LTRs are identical at the time of integration. We then clustered the anchors and excluded those clusters derived from unfixed SVA elements, present in regions with abnormally high coverage, or within 200 nt of a HK2 or a related locus in the reference genome (namely, RepeatMasker regions HERVK, HERVK14C, HERVK3, HERVK9, LTR5, LTR5_Hs, LTR5A, or LTR5B).

In the second step, we collected all reads in the BAM file both that did not map perfectly to the reference genome (according to their compact idiosyncratic gapped alignment report [CIGAR] value) and that had an 8-nt match to the start or end (sense and antisense) of the K113 LTR. We removed this 8-nt sequence and the following sequence and then remapped the resulting trimmed reads to the genome sequence using the Novoalign program (Novocraft Technologies). In all instances where the coordinates of these trimmed read clusters were close to those of the (filtered) RetroSeq clusters described above (Fig. 1), we moved to the final, validation stage. A second example of this mapping of RetroSeq and trimmed read clusters is shown in Fig. S4 in the supplemental material.

**FIG 2:**
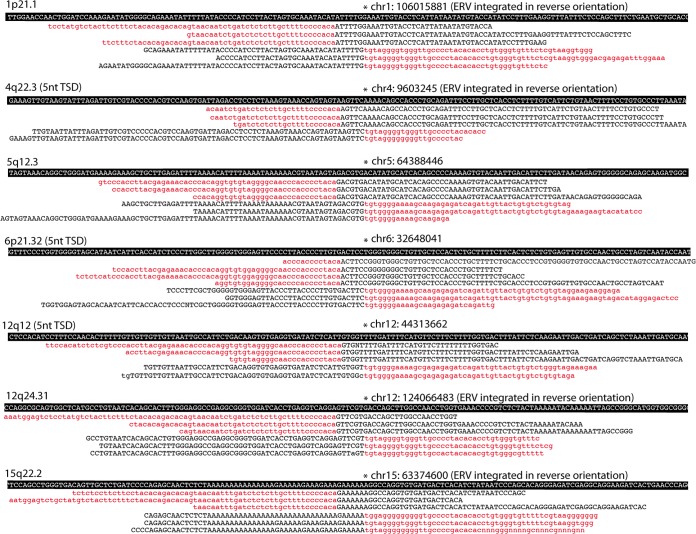
Validation of integrations. Edited output of the BreakAlign program showing a few representative chimeric NGS reads that span the integration site of unfixed loci. In each read, part of the sequence is viral (red lowercase nucleotides) and the other part aligns to the reference preintegration sequence shown above (on a black background). For each locus, we have chimeric reads from upstream and downstream flanks of the integration, both of which contain the 5-nt-long or (unless indicated) 6-nt-long target site duplication (TSD). Loci found in TCGA patients that are not shown here are described by Marchi et al. (33).

**FIG 3:**
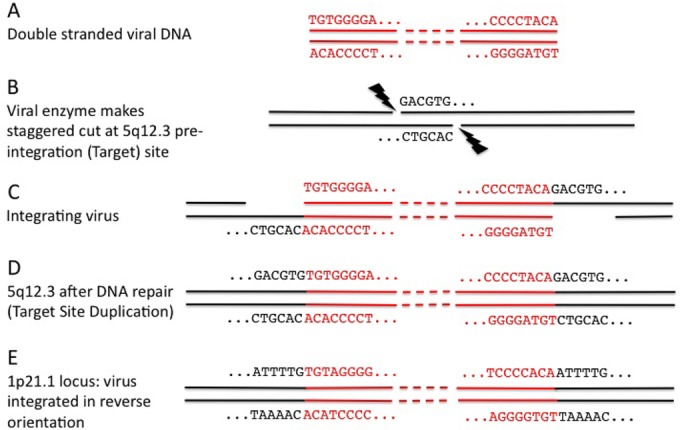
How chimeric reads result from ERV integration. (A to D) A guide to interpretation of outputs by use of locus 5q12.3 as an example. After reverse transcription, viral double-stranded DNA (red) is integrated into the chromosome. The viral integrase enzyme makes a staggered cut, typically of 6 nt, into which the viral DNA is inserted. DNA repair of the now single-stranded DNA on either side of the integration produces six identical nucleotides (the target site duplication) flanking the virus. (E) However, in some cases the virus has integrated in reverse orientation, and an example of where this has occurred is shown for locus 1p21.1. Note the changed viral sequence.

In the third step, we confirmed the presence of an integration at the coordinates described above by finding chimeric reads using our own Perl script (the BreakAlign program). This uses the BLASTN program (27) to align reads to the region of the reference genome sequence, typically ∼200 nt, that spans our combined RetroSeq plus trimmed read clusters. Examples of BreakAlign outputs are shown in Fig. 2, and a guide to their interpretation is provided in Fig. 3. Chimeric reads that span an ERV integration are output with the part of the read that aligns to the genome sequence in uppercase and the part of the read that is from the virus in lowercase. In most cases we found multiple reads spanning both ends of the integration and showing the target site duplication, which results from the staggered cut made in the host double-stranded DNA by the viral integrase enzyme.

### (ii) Pipeline for WGS500 genomes.

We measured the frequency of all loci found in the analysis of TCGA genomes described above and others reported in the literature (21) in 332 WGS500 patients. For each locus we constructed two artificial chimeric sequences representing the start and the end of the integration. Each chimeric construct was 100 nt in length and contained 50 nt of the LTR and 50 nt of the flanking genome sequence. The chimeric constructs were then scanned against all the reads in the WGS500 data set using the BLAT program (28) with a minimal identity threshold of 0.8 and a length of alignment of 90 nt. The number of matches to the chimeric construct was recorded, and the locus was classified as absent in the case of zero matches and present if there were one or more matches.

We also examined 46 WGS500 patient genomes for previously unknown loci in regions at least 100 nt from a repeat using the RepeatMasker coordinates. This corresponds to 37% of the human genome sequence (see above). In the first step, we scanned all sequenced reads against the K113 LTR using BLAT with a minimum identity threshold 0.8. Chimeric reads where at least 20% of their length was from the LTR were selected, and the LTR part was trimmed off. The resulting sequences were then mapped back to the human genome (version GRCh37d5/hg19d5, referred to as hg19) using BLAT with a minimum identity threshold of 0.99. The set of potential integration points was compared to the RepeatMasker coordinates to remove all sequences within 100 nt of a known repeat element. The remaining reads were analyzed using BreakAlign, as described above, but revealed no additional loci.

### An expected number of loci.

The copying of ERVs creates new loci that are present initially in a single host individual. We expect the frequency of these loci to increase in the host population only as a result of genetic drift. A few ERV loci have been co-opted (13) and in theory might have gone to fixation as a result of positive selection, but we assume that this is rare.

We can calculate the number of loci that we expect to be unfixed within a given sample size (i.e., present in some but not all the individuals in that sample) using the infinite-sites model of genetic drift (29). Here we assume that the integration of ERVs can be treated in the same way as point mutations. This model allows an unlimited number of unique sites into which the ERV can integrate (forming loci) without reversals to the preintegration state, of which no examples are known (both solo LTRs and full-length loci are detected by our mining).

$$E(S)=\theta \overset{n-1}{\underset{i=1}{\sum }}\frac{1}{i}$$

In the equation presented above, *E*(*S*) is the expected number of segregating sites (unfixed loci) in a sample of *n* alleles (genomes). In our study, *n* was equal to 53 (26 diploid individuals plus the reference sequence). θ is the average heterozygosity for pairs of randomly chosen alleles (genomes) and equals 4*N_e_*μ, where *N_e_* is the long-term effective population size (taken as 10,000) and μ is the mutation (integration) rate (29). We calculated μ by dividing the number of human-specific insertions (i.e., loci represented by preintegration sites in the chimpanzee, which is 113) by the number of generations in the human lineage since divergence, assuming an average generation time of 20 years and the human-chimpanzee divergence to be 6 million years ago (30). Some loci would have integrated before the divergence of the human and chimpanzee; i.e., they were unfixed in the common ancestor and became fixed in the human and lost in the chimpanzee (lineage sorting). Our estimate of the average time for a neutral allele to go to fixation in the common ancestor of the human and chimpanzee is 800,000 years (4*N_e_* generations), so we added this to the divergence date. Our value of θ (which is equal to 4*N_e_*μ) is therefore 13.3. *i* is the summation variable.

We cannot simply use the expectation from the equation presented above because our genome mining does not reveal unfixed loci that are present in the reference genomes. We therefore used the simulation of the infinite-sites model in the program ms (31), which generates samples drawn at random from a population that obeys the Wright-Fisher model of genetic drift and an infinite-sites model of mutation. These samples take the form of a presence/absence matrix of samples (genomes) and alleles (loci). We randomly selected one of these samples to represent the human genome sequence and counted only loci that were represented by a preintegration site in this sample. This number represents the recovery of loci in our mining procedure. Both the equation and the simulation gave an expectation that 60 loci would be unfixed in our sample of 26 patients, but the simulation showed that 13 of these loci would also be in the reference sequence and thus not observed by our mining method. This value of 13 is consistent with that determined in our previous study, where we found 8 loci in the reference to be unfixed in a smaller sample size of only 19 individuals screened by PCR, and an extrapolation to include untested loci raised this value to 11 (30).

In the simulation, we calculated the mean number of loci from 1,000 runs. The variance depends on the level of recombination, and we incorporated free recombination by summing in each run the results from 10,000 coalescent trees, on each of which the insertion rate was 0.0001μ. This ensured that no two loci appeared on the same coalescent tree.

### Limitations of the model.

We used the only available method for measuring the integration rate of HK2, which was to compare the reference human and chimpanzee genomes. We therefore assume that these are single haploid genomes. However, there might have been a bias toward including or excluding transposable elements that were unfixed within the individuals whose genome data were used to construct the human reference sequence. We cannot find any statement in the literature indicating that such polymorphisms were encountered or any description of the procedures used to resolve them. It is therefore possible that the human reference genome contains loci from several individuals, which would inflate our integration rate and, hence, the number of loci expected within a sample of individuals. Also, by possibly comparing individuals against a multiple-individual reference human genome, we are likely to find a lower number of loci per individual (one of our assumptions is that by comparing genomes against the reference, we compare the genomes of only two individuals). Thus, we can consider that the number of loci observed in our sample is a conservative minimum number.

The divergence time from the chimpanzee (6 million years ago) that we used is near the upper boundary of published estimates, and the generation time is toward the lower boundary of published estimates (32). Use of even an early date of 7 to 13 million years ago for the divergence of humans and chimpanzees, recently inferred by measuring current generation times and generational mutation rates in the Great Apes (32), only approximately halves the integration rate. This halving has the same effect in the infinite-sites model as halving the long-term effective population size from 10,000 to 5,000 or halving the generation time from 20 to 10 years. All three adjustments on their own still give a significantly higher expectation of 24 loci in our simulation with the ms program (*P* < 0.01).

To simulate the effect of the lineage ceasing to copy at different points in our evolutionary past, we adjusted the ms program. The original program has two main steps. First, it generates random genealogies for a specified number of samples in which branch length is measured by the number of generations (1 unit = 4*N_e_* generations). It then uses a Poisson distribution to randomly place mutations (which in our case represent integrations) onto these branches. We simulated a cessation of copying by excluding those integrations that the program would have placed within a certain distance from the tree tip; e.g., to simulate a cessation at 1 million years ago, we excluded integrations within 1.25 units of the tree tip. For each simulated cessation time, we increased the integration rate inferred from the number of human-specific loci to reflect the shorter period of time in which these integrations would have occurred.

Loci might have been under negative selection. Although no pathological effect has been established for ERVs in humans, there are examples in other animals (7), and retroviruses are inherently oncogenic due to their copying mechanism. If we assume that all loci are under negative selection (even those that have gone to fixation), then we need to increase the integration rate to end up with the observed number of human-specific loci. This will, in turn, increase the expected number of loci within our sample (see the simulation in Methods and Fig. S3 in the supplemental material).

## RESULTS

### Unfixed loci.

Our search for HK2 loci in 26 patients of The Cancer Genome Atlas (TCGA) project revealed 13 loci that are not in the reference sequence (Table 1). In Fig. 2 we show the output from the final, confirmatory stage of our mining pipeline: the alignments of chimeric NGS reads to the preintegration site in the reference human genome (see Fig. 3 for guidance on interpretation). One of these loci is the well-studied, but quite rare, locus K113. On average, each individual had six loci that were not in the hg19 version of the human reference genome, and we found that, as expected, these unfixed loci were often in the heterozygous state (see Table S1 in the supplemental material). The frequency of the presence of these 13 loci in 332 WGS500 patients ranged from only one individual to over 95% of them (Table 1). Another study (21) presented 31 putative loci (see Table S6 in the supplemental material for reference 21). However, only 15 of these are genuine unfixed loci; the other 16 represent a variety of mining artifacts (see the supplemental material). Eleven of these 15 loci were present in our TCGA patient genomes, and the other 4, all of which were present at low frequencies (below 15%) in that other study (21), were found to be present in our larger WGS500 sample. Interestingly, of this combined total of 17 loci, we recently reported (33) that 8 were present among the loci identified in two fossil hominins (34). Two of these eight were also recently recovered from modern humans (35).

**TABLE 1:** The 17 HK2 loci that are not in the human reference genome^*j*^

Cytoband	Coordinate^*a*^	Other name(s)	Flanking region	Frequency		
				TCGA (*n* = 26)	WGS500 (*n* = 332)	Lee et al. (*n* = 44^*b*^)
1p21.1	106015874–106015881		—^*c*^	0.04	0.003	0
1p13.2^*d*^	111802591–111802598	DE5, ERVK1	L1	0.62	0.593	0.35
1q41	223578303–223578310	ERVK2	L1	0	0.006	0.02
4q22.3	9603239–9603245	ERVK6	ERV	0.96	0.958	0.86
5q12.3	64388439–64388446	ERVK9	L1	0.15	0.075	0.12
5q14.1	80442265–80442272	DE6, NE1, ERVK10	RASGRF2 intron	0	0.093	0.14
6p21.32	32648035–32648041^*e*^		L1	0.46	0.443	0
6q26	161270898–16127090	DE2, ERVK12	—^*c*^	0.96	0.834	0.70
9q34.11	132205208^*f*^	DE7, ERVK16	MaLR	1.00	0.961	0.33
11q12.2	60449889^*f*^	DE4, ERVK18	L1^*g*^	0	0.003	0.02
12q12^*h*^	44313656–44313662	ERVK20	L1 in TMEM117 intron	0.31	0.241	0.14
12q24.31	124066476–124066483	ERVK21	Alu^*g*^	0.35	0.238	0.14
13q31.3	90743182–90743189	NE2, ERVK22	AT rich^*g*,*i*^	0.15	0.190	0.12
15q22.2	63374593–63374600	ERVK24	Alu	0.81	0.889	0.79
19p12	21841536–21841542	K113, DE1, ERVK26	—^*c*^	0.08	0.087	0.08
19q12^*d*^	29855781–29855787	DE3, ERVK28	—^*g*^	0.54	0.678	0.56
20p12.1^*h*^	12402386–12402392	ERVK30	—^*c*^	0	0.015	0.05

a The 5- or 6-nt difference between coordinates is the length of the target site duplication (hg19).

b From 41 germ line genomes from cancer patients plus 3 healthy individuals from the HapMap project (21).

c Single-copy nontranscribed DNA region.

d Also found by Kahyo et al. (35).

e Locus present in some publicly available HLA haplotype sequences.

f We found evidence for only one side of the integration.

g Within long noncoding RNA.

h As validated by PCR by Lee et al. (21), one integration and one preintegration site for both loci.

i The locus is also 12 nt from an Alu.

j The distribution of loci among individuals and zygosity in the 26 TCGA patients are given in Table S1 in the supplemental material. Cytobands are taken from http://www.tallphil.co.uk/bioinformatics/cytobands.

We found no examples of somatic copying (loci in the cancer genome that were not in the corresponding germ line genome of the same patient), and all loci were found in more than one individual.

### Is the lineage still replicating?

A neutral population genetic model predicted the presence of 47 loci in our sample of 26 individuals, while we recovered only 13. One explanation for this difference is that we have overlooked loci. In the absence of a proper control, two facts suggest that our approach has performed well. First, loci that are within other repeats, such as LINE and Alu repeats, are much more difficult to find than loci in single-copy DNA regions because there are many similar copies of the integration site. If we had missed loci, we would expect these to have been within repeats. However, as shown in Table 1, a large proportion of our previously unknown loci (8 out of 13) were within repeats. If we compare this to the HK2 loci in the human reference genome (hg19), we find that 439 out of 987 loci are in repeats according to the RepeatMasker (36) coordinates. Second, the total number of loci that we found is also similar to our reanalyzed findings of another study (21): 13 versus 15 loci, respectively. That study appears to have missed one common locus that was in a repeat and one rare locus, but it appears to have found four additional rare loci in its slightly larger sample size.

**FIG 4:**
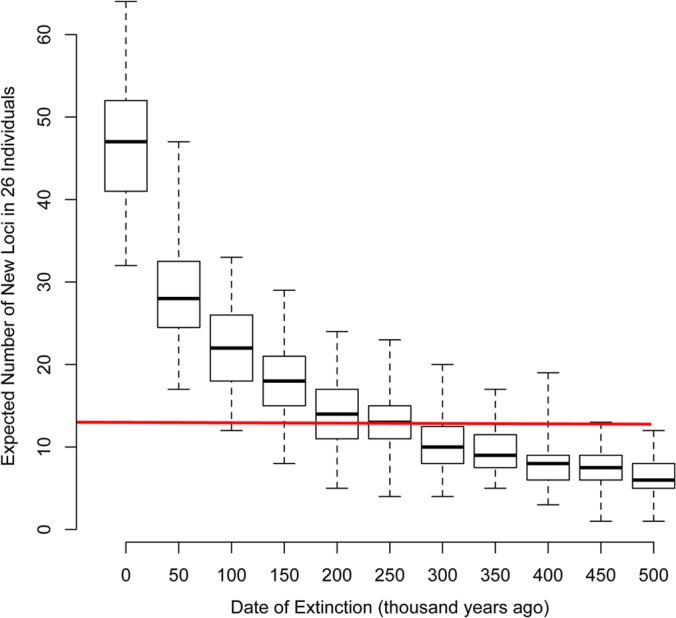
Comparison of the observed and expected numbers of loci. The number of loci in the 26 TCGA patients predicted by the genetic drift model is shown. Along the *x* axis are the expectations assuming either that the rate of copying until the present day is constant (the date of extinction is year 0) or that the copying of loci ceased at different dates in the last half million years. The red line across the figure shows the observed number (*n* = 13). The boxes show the medians, interquartile ranges, and the most extreme values from 10,000 replicates.

Another possible explanation for our finding so few loci is that the integration rate of the lineage has significantly decreased or even stopped. Assuming that the rate that we used is not significantly biased (see “Limitations of the model” above) and that the observed number of 13 loci is a conservative (lower-bound) estimate, then, modeling a possible cessation of copying, the difference between an observed 13 and an expected 47 suggests that the lineage was active until at least 250,000 years ago (Fig. 4). Due to the limitations of our study, we cannot be sure if germ line replication continues today.

## DISCUSSION

Our study, combined with the findings of the analysis of Subramanian et al. (4) of loci in the human reference genome, now provides the necessary coordinates for a PCR-based screen to examine HK2 polymorphisms in the human population. Each locus can exist in several states within the human population: full-length provirus, a solo LTR, and a preintegration (empty) site. The full-length provirus can also be polymorphic for coding capacity; e.g., locus 22q11.21 has a full-length ORF in the Tera-1 cancer cell line (37) but not in the reference genome (4). Specifically, we now need to identify the loci capable of producing proteins and measure the frequency of their full-length ORFs. A recent PCR-based study (35) found that loci 1p13.2 and 19q12 in our study were represented by solo LTRs in 512 and 558 individuals, respectively, with 288 and 242 individuals, respectively, having preintegration sites and no individual having full-length proviruses. Both of these are old loci, being found (and identified as loci De5 and De3, respectively) in an archaic human fossil belonging to a lineage that diverged from modern humans ∼400,000 years ago (33). Some of the 10 loci in the reference genome that are known to be unfixed (35) also appear to be represented solely by the solo LTR (30, 38). RNA transcripts from loci in the reference sequence have already been identified (15, 39–41). When the polymorphisms among the HK2 loci have been characterized, the transcriptional activity of each potentially protein-expressing locus (both fixed and unfixed) could be measured by transcriptome sequencing. Investigations into the potential of HK2 in immunotherapy might then be able to take into account variation caused by differences between individuals in their protein-coding loci.

All the loci were found in more than one individual, so there was no evidence of somatic copying in cancer genomes. Only one example of somatic copying involving HK2 has been reported, and this appeared to be LINE mediated rather than a true integration (21). However, the sample sizes in both studies were quite small, and unless the copying event happened in the founder cancer cell or within the first few rounds of replication, such loci would be in only a minority of cells and, hence, NGS reads from a tumor. We note that somatic copying could occur in the absence of intact loci in the germ line: replication-competent loci can be generated by recombination between defective loci. This has been shown to occur in immunodeficient mouse ERVs (42) and has been inferred by analysis of HK2 sequences derived from the plasma of HIV-infected patients (43). Furthermore, two research groups have independently constructed a replication-competent virus, albeit of limited infectivity, from the consensus sequences of recently integrated, though defective, HK2 loci (44, 45). In NGS, detection of somatic copying by transposable elements such as HK2 will be helped by target enrichment using the flanking regions of the element; e.g., see the work of Iskow et al. (23).

The results of our earlier investigation into insertional polymorphism in HK2 (30) were not inconsistent with the predictions of the same neutral population genetic model. Our earlier study used a less powerful test: only loci in the reference genome that were found to be unfixed within a PCR screen of genomic DNA from 19 individuals were counted. The only other data relevant to the question of whether or not these viruses are copying in the germ line of the present-day human population are the integration dates of individual loci inferred from nucleotide substitutions. The paired LTRs of an ERV locus are identical at integration and accumulate substitutions at the host background level, thus gradually diverging from each other over time. Unfortunately, although some loci have identical LTRs (20), they might still be almost a million years old, given a background rate of ∼1 × 10^−9^ substitutions per site per year. Another approach to dating ERV integrations is to use the sequence divergence between homologous copies of the same locus in different individuals (estimating the time to the most recent common ancestor). The most recent integration age of a locus estimated using this method and a plausible substitution rate of 1.3 × 10^−9^ (46) is 150,000 years (47). This is consistent with our findings (Fig. 4). Our new analysis suggests that the HK2 lineage was copying within the human population until at least 250,000 years ago. We also emphasize that the repeat regions of the human genome largely remain terra incognita. For example, an HK2 virus integrated into a centromeric repeat in our common ancestor with the chimpanzee and was subsequently copied around our genome by that repeat at least 100 times. This provirus (K111) is not in the reference sequence and has only just been discovered by PCR-based analysis (48). Thus, due to the limitations of our population model, the quality of the existing human reference genome, and the available sequencing technologies, we consider that this is a conservative estimate of the population-level replication of HK2. Whether it ceased copying after 250,000 years or the observed pattern is a result of current limitations will be resolved in the next few years with the increasing availability of complete genome sequences and the increased ease of detection that will come with the longer reads emerging from NGS technologies. We need to estimate the rate of HK2 integration using genomes derived from single individuals (rather than the current composite) and find all insertionally polymorphic loci (not just ones absent in a reference sequence) across ∼200 individuals from different ethnic groups (the theoretical expectation levels off in much larger samples).

We found two loci within introns. One of these loci (5q14.1), which had been found in the Denisovan and Neandertal genomes (De6 and Ne1, respectively), is integrated within the *RASGRF2* gene. This gene has been linked with the regulation of the dopamine neuron activity in the mesolimbic pathway, more specifically, with the response to reward, and effects on alcohol use and abuse (14). We can hypothesize that this unfixed locus might have an effect on reward phenotypes and the propensity to addiction, but further studies are needed to investigate this. It is now possible to test for associations between disease states and unfixed HK2 loci, both the 17 described here and the 10 loci in the reference genome that are known to be unfixed (35). Our study brings to the surface the possibility that unmapped polymorphisms of repetitive elements might be playing a role in disease phenotypes. Population-wide mining for such polymorphisms should help establish genetic associations of unfixed loci and disease.

## Supplementary Material

Supplemental material
