# Supplementary material for: Unfixed Endogenous Retroviral Insertions in the Human Population
Source: J Virol. 2014 Sep;88(17):9529–37. doi: 10.1128/JVI.00919-14 (PMC4136357; doi:10.1128/JVI.00919-14)
Supplement: Supplemental material [file supp_88_17_9529__index.html]

Unfixed Endogenous Retroviral Insertions in the Human Population — Supplemental material 

# Unfixed Endogenous Retroviral Insertions in the Human Population

## Supplemental material

**Files in this Data Supplement:**

- Supplemental file 1 -

  Supplemental methods

  Fig. S1 (False-positive clusters caused by SVAs.)

  Fig. S2 (Abbreviated output from BreakAlign script.)

  Fig. S3 (Single illustrative examples of toy Wright-Fisher simulation.)

  Fig. S4 (Genome Browser screenshots for region of locus 6q26, showing results 121 from one patient with the integration and from one patient without it.)

  PDF, 1.8M
