# Supplementary material for: Unfixed Endogenous Retroviral Insertions in the Human Population
Source: J Virol. 2014 Sep;88(17):9529–37. doi: 10.1128/JVI.00919-14 (PMC4136357; doi:10.1128/JVI.00919-14)
Supplement: Supplemental material [file JVI.00919-14_zjv999099395so1.pdf]

Unfixed endogenous retroviral insertions in the human population

Emanuele Marchi, Alex Kanapin, Gkikas Magiorkinis and Robert Belshaw

Supplementary Methods

Common sources of 'false positives' in mining NGS whole genomes

The non-autonomous transposable element called an SVA contains nucleotides 1-329 and 799-927 of the K10 LTR (1). *RepeatMasker* annotates either 3425 (hg18) or 3733 (hg19) regions as being SVAs. Insertional polymorphisms among SVAs are common and produce many false positives when analysed only with *RetroSeq* (in 'Discovery' mode). These false positives appear as one-side clusters with the anchor read (see main text) downstream of the SVA integration (the intervening non-LTR region of the SVA is apparently too long to create corresponding upstream anchors). In Fig. S1 panels A-E we show a representative series of clusters in which the mate reads (see main text) have all been shown to match K113 by BLAST. We see that the matching part of most reads end shortly past position 300 in the K113 LTR (as expected given that 1-329 region of the LTR is known to be within the SVA). Interestingly, we do not find matches to the smaller second fragment of the LTR, namely 799-927, which in the SVA is downstream of the larger fragment and close to the polyA tail. The presence of this polyA tail was confirmed in all the examples shown except for one (B), and we confirmed the absence in all examples of LTR regions outside the two regions known to be in the SVA. We note that *RetroSeq*, run a second time in 'Call' mode, would exclude such one-sided clusters, but our mapping of all *RetroSeq* clusters to the trimmed-read clusters (see main text) allows us to detect integrations where one side had been truncated. The presence of HK2 fragments within SVAs has led to them being mistakenly identified as unfixed HK2 loci in the literature: in Fig. S1 panel F we show the sequences mistakenly attributed to an HK2 locus called ERVK31 (2).

Some other apparent insertional polymorphisms result from polymorphism in the degree of ERV fragmentation. One example is on chromosome 15 at position 28430104 (hg19), where there is only a 23 long nucleotide fragment of the 3' end of a 3' (+ve sense) LTR. This fragment is too small to be recognised by many automated searches, but in some TCGA genomes we find more complete LTRs. Where there is a longer LTR fragment, these are recognised erroneously by *RetroSeq* as novel integrations. In Fig. S2 we show this fragment, with flanking genomic regions, aligned to chimaeric reads by our *BreakAlign* script. This type of false positive was only revealed by visual inspections of the *BreakAlign* outputs. In the Lee at al. study (2), the purported new unfixed locus ERVK23 is derived from this fragment, which is erroneously identified as the TSD (Fig. S2). Their positive clipped contig, which should contain the end of the LTR, is instead the contiguous upstream 3' LTR region. Their negative clipped contig does not derive from the same locus but contains instead an internal fragment of a 5' LTR integrated in the opposite orientation. Purported locus ERVK11 is similarly derived from an LTR in the human genome reference sequence, in this case one that belongs to an older clade of HK2 integrations, LTR5B (3), which are not human-specific. The LTR at this coordinate contains a 13 nt internal insertion compared to the LTR5B reference sequence, and this deletion is apparently mistaken by the Lee et al. searching algorithm for the TSD. Of the remaining 'false positives' in that study, ERVK13, 17, and 29 appear to be generated by the presence of HK2 fragments in the reference genome at the reported coordinate, and ERVK3, 4, 5, 7, 8, 14, 15, 19, 25 and 27 by short (<= 16 nt) sequence matches or matches to the older LTR5B clade of HK2 mentioned above.

Toy simulation showing effect of selection on expected number of loci

To investigate the effect of negative selection on our neutral population genetic model we ran a toy simulation in the R programming language. This followed a Wright-Fisher model of genetic drift in a population of 100 haploid individuals over 2000 generations with an integration (mutation) rate of 0.01 new loci per individual per generation. At the end of the run, the total number of loci present in a random sample of 10 individuals was calculated after excluding those loci that are present a randomly selected single individual (taken here to represent the reference genome). These are

the 'new' loci that are equivalent to the 13 loci we have found in our examination of the 26 TCGA genomes.

The above simulation (Fig. S3 panel A) resulted in a mean of 5 new loci, with 20 loci accumulating in the reference (= all the fixed loci plus unfixed loci with probability equal to their frequency). We then introduced negative selection into the simulation and simultaneously increased the integration rate to ensure that the number of loci appearing in a single reference genome remained constant at 20 (Fig. S3 panels B and C). This adjustment of the integration rate can be viewed as compensating for the loss of loci by selection. We then measured the mean number of 'new' loci from 1000 runs of the simulation. As shown in Fig. S3, the number of new loci increases with increasing levels of negative selection.

Supplementary References

1. Ono M, Kawakami M, Takezawa T. 198. A novel human nonviral retroposon derived from an endogenous retrovirus. *Nucleic Acids Res.* **15**:8725–8737.

2. Lee E, Iskow R, Yang L, Gokcumen O, Haseley P, Luquette LJ, III, Lohr JG, Harris CC, Ding L, Wilson RK, Wheeler DA, Gibbs RA, Kucherlapati R, Lee C, Kharchenko PV, Park PJ, Cancer Genome Atlas Research Network. 2012. Landscape of somatic retrotransposition in human cancers. *Science* **337**:967–971.

3. Subramanian RP, Wildschutte JH, Russo C, Coffin JM. 2011. Identification, characterization, and comparative genomic distribution of the HERV-K (HML-2) group of human endogenous retroviruses. *Retrovirology* **8**:90.

Supplementary Figure Legends

Fig. S1. False positive clusters caused by SVAs. Panels A-E are screenshots from the NCBI BLAST website showing the mate sequences in five different clusters each aligned to the full length K113 LTR. Matches all correspond to sections of the HK2 LTR that are known to be within SVAs. These are all unfixed SVAs that are not in the reference genome sequence. Panel F shows an SVA previously reported as an unfixed HK2 locus (see text).

Fig. S2. Abbreviated output from *BreakAlign* script. Viral regions are in red. Here the small LTR fragment that is in the human reference sequence is aligned both to the longer LTR sequences within some of our chimeric NGS reads (reads 1-6) and to the sequences for ERVK23 in table S6 of Lee at al. (2). Nucleotides in upper case match the reference; nucleotides in lower case do not match the reference.

Fig. S3. Single illustrative examples of toy Wright-Fisher simulation. A-C show results with three different levels of negative selection. Graphs show both the number of fixed loci, which gradually increases, and the total number of unfixed loci that are present in each generation, which soon reaches an equilibrium.

Fig. S4. IGV Genome Browser screenshots for region of locus 6q26, showing results from one patient with the integration and from one patient without it. The four panels are, in descending order, reads from the cancer genome, reads from the germline genome, trimmed reads, and anchors (see main text) whose mate matches K113. A) This is an individual homozygous for the integration. B) This is the single individual in Table S1 that is homozygous for the pre-integration site. Note the absence of

trimmed reads mapping to this region (third panel) and absence of mate reads that match K113 (bottom panel). The colored reads in the first two panels are anchors whose mates map to other genomic regions but do not match K113

Fig. S1

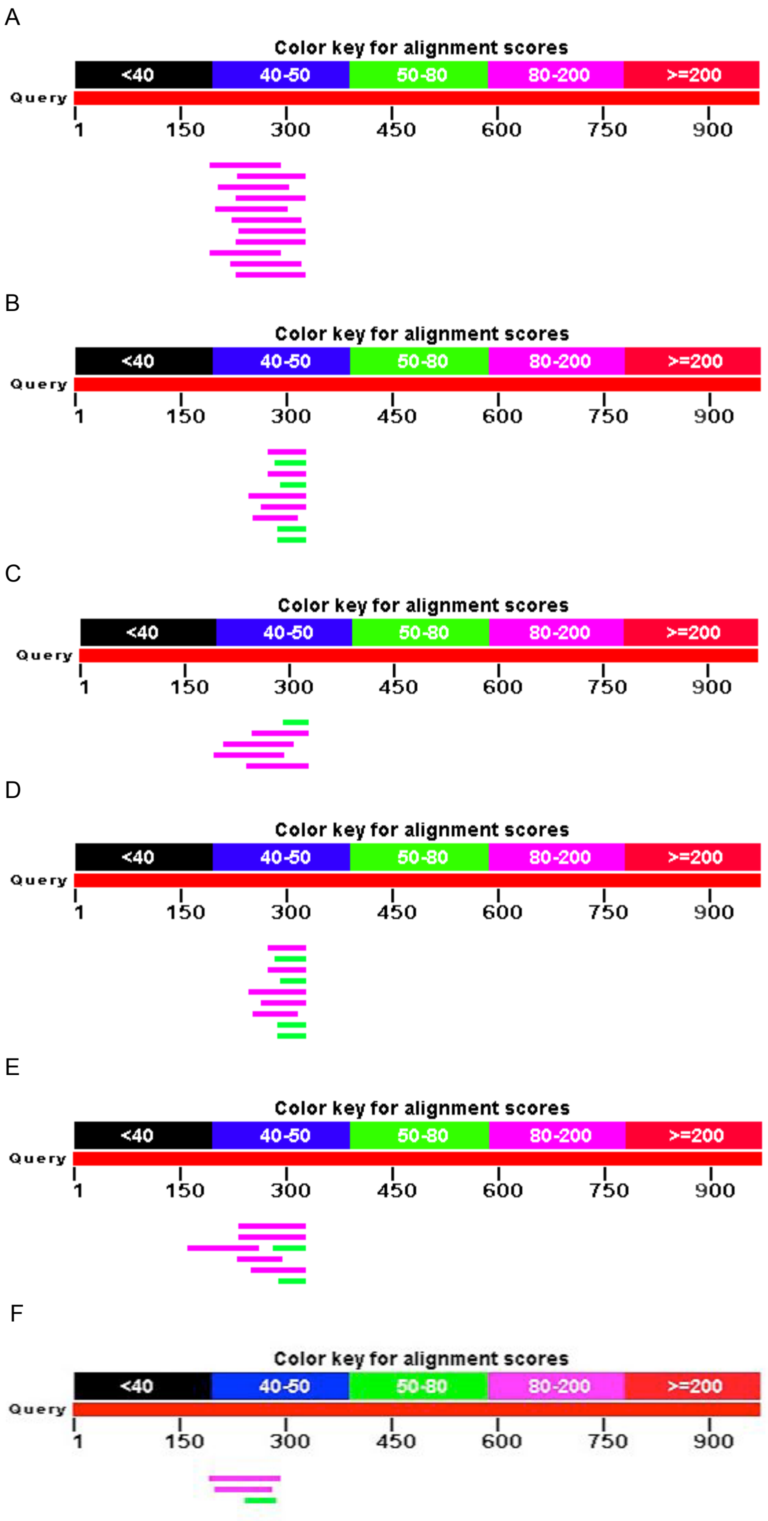
A
Color key for alignment scores
<40
40-50
50-80
80-200
>=200
Query
1
150
300
450
600
750
900
B
Color key for alignment scores
<40
40-50
50-80
80-200
>=200
Query
1
150
300
450
600
750
900
C
Color key for alignment scores
<40
40-50
50-80
80-200
>=200
Query
1
150
300
450
600
750
900
D
Color key for alignment scores
<40
40-50
50-80
80-200
>=200
Query
1
150
300
450
600
750
900
E
Color key for alignment scores
<40
40-50
50-80
80-200
>=200
Query
1
150
300
450
600
750
900
F
Color key for alignment scores
<40
40-50
50-80
80-200
>=200
Query
1
150
300
450
600
750
900

Fig. S2

```
                                                          * chr15: 28430104
TCATTTTAGTAGCTACTGAGTAAGAAAATGCTAATCTATTGTAGGGGCAACCCACCCCTACAATCTATAA reference sequence (hg19)
         gtcccaccttacgagaaacacccacaggtgTGTAGGGGCAACCCACCCCTACAATCTATAA read 1
         gtcccaccttacgagaaacacccacaggtgTGTAGGGGCAACCCACCCCTACAATCTATAA read 2
       tcgtcccaccttacgagaaacacccacaggtgTGTAGGGGCAACCCACCCCTACAATCTATAA read 3
         gtcccaccttacgagaaacacccacaggtgTGTAGGGGCAACCCACCCCTACAATCTATAA read 4
     tctcgtcccaccttacgagaaacacccacaggtgTGTAGGGGCAACCCACCCCTACAATCTATAA read 5
       tcgtcccaccttacgagaaacacccacaggtgTGTAGGGGCAACCCACCCCTACAATCTATAA read 6
 ...ctctcgtcccaccttacgagaaacacccacaggtg                                ERVK23 Positive clipped contig
                                       TGTAGGGGCAACCCACCCCTACA         ERVK23 'TSD'
```

Fig. S3

A) Neutral. Mean number of new loci in sample = 4.8

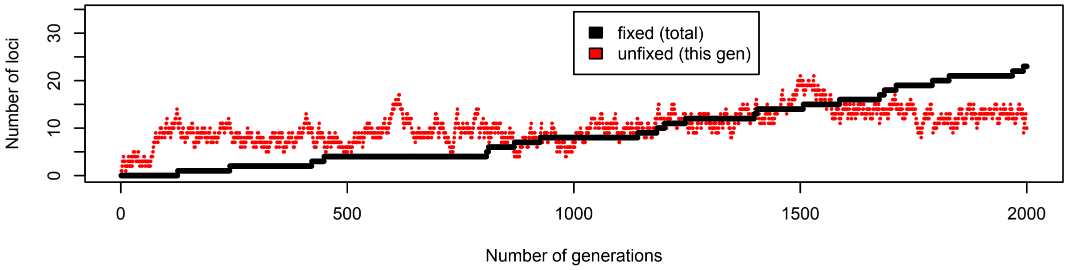

B) Weak negative selection (s = 0.001). Mean number of new loci in sample = 5.8

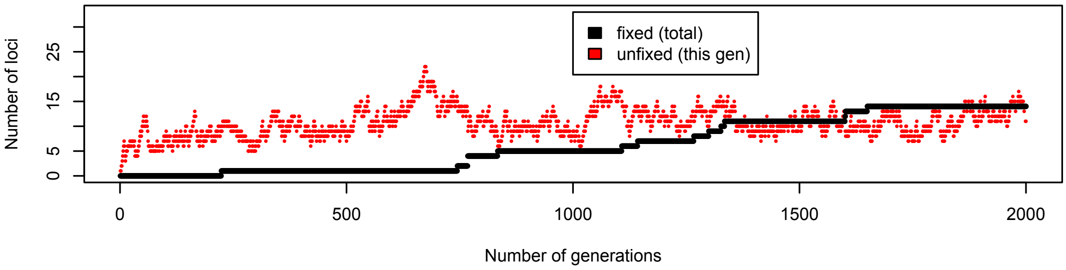

C) Strong negative selection (s = 0.01). Mean number of new loci in sample = 40.5

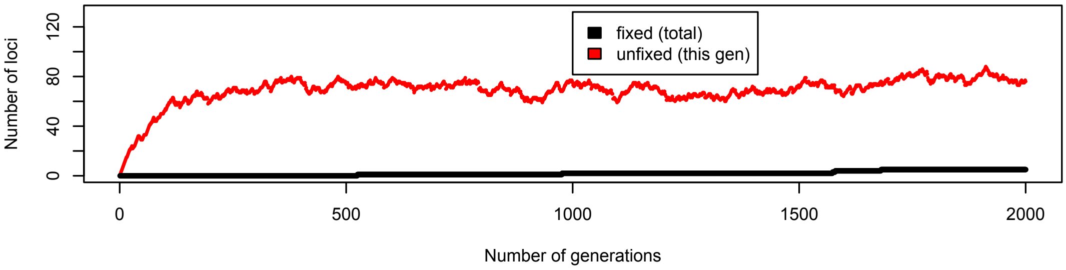

Fig. S4

A)

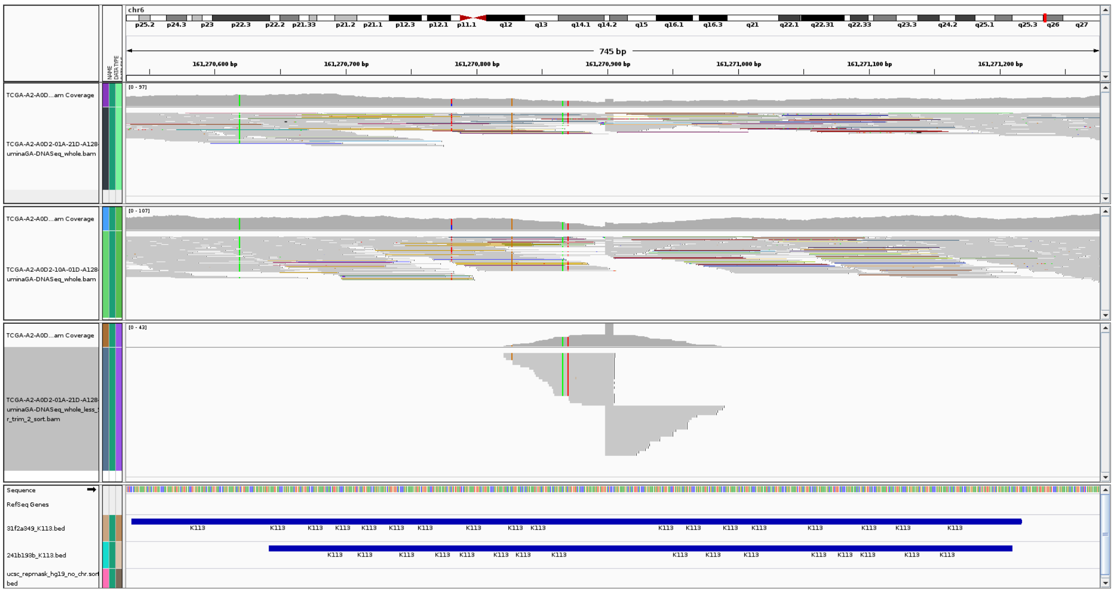
chr6
745 bp
161,270,600 bp
161,270,700 bp
161,270,800 bp
161,270,900 bp
161,271,000 bp
161,271,100 bp
161,271,200 bp
TCGA-A2-A0D...am Coverage
TCGA-A2-A0D2-01A-21D-A128 uminaGA-DNASeq_whole.bam
TCGA-A2-A0D2-10A-01D-A128 uminaGA-DNASeq_whole.bam
TCGA-A2-A0D2-01A-21D-A128 uminaGA-DNASeq_whole_less_r_trim_2_sort.bam
Sequence
RefSeq Genes
31f2a349_K113.bed
241b193b_K113.bed
ucsc_repmask_hg19_no_chr.sort.bed

B)

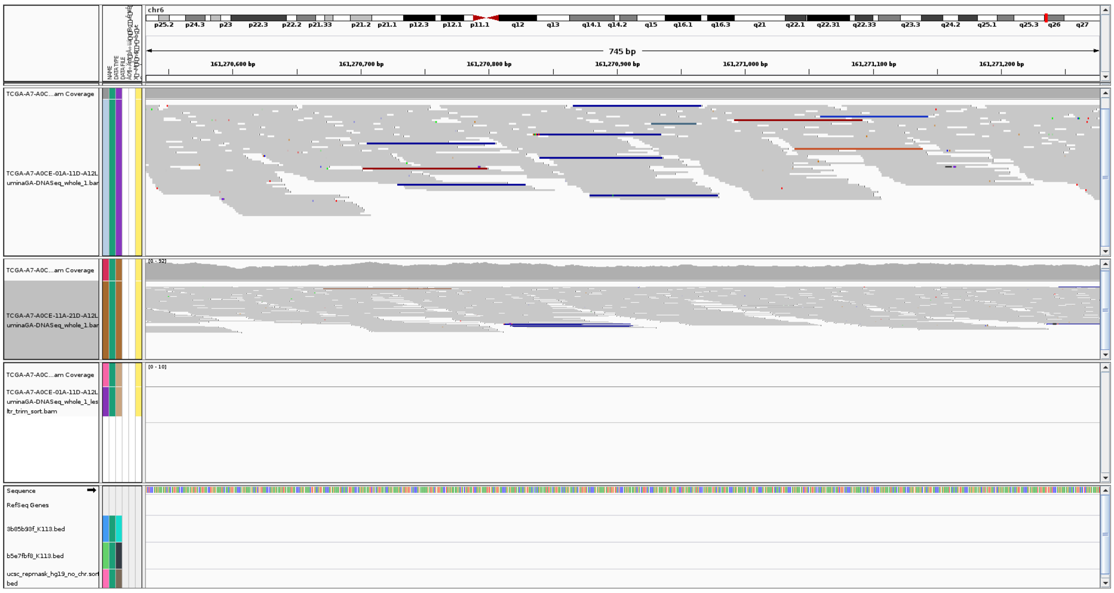
chr6
745 bp
161,270,600 bp
161,270,700 bp
161,270,800 bp
161,270,900 bp
161,271,000 bp
161,271,100 bp
161,271,200 bp
TCGA-A7-A0C...am Coverage
TCGA-A7-A0CE-01A-11D-A12L uminaGA-DNASeq_whole_1.bam
TCGA-A7-A0CE-11A-21D-A12L uminaGA-DNASeq_whole_1.bam
TCGA-A7-A0CE-01A-11D-A12L uminaGA-DNASeq_whole_1_less_ltr_trim_sort.bam
Sequence
RefSeq Genes
3b05b93f_K113.bed
b5e7fbf8_K113.bed
ucsc_repmask_hg19_no_chr.sort.bed

Table S1. Distribution of loci among the 26 TCGA patients. 0 = absent; 1 = present (zygosity given if confidently known: hom = homozygous; het = heterozygous).

Locus	Ovarian cancer						Breast cancer										Lung cancer					Brain cancer				
1p21.1	0	0	0	0	0	0	0	1[a]	0	0	0	0	0	0	0	0	0	0	0	0	0	0	0	0	0	0
1p13.2	1-het	0	1-het	0	0	0	1-het	1-het	1-hom	1-het	1-het	0	1	0	1-hom	0	1	1-hom	1-het	1-het	1-hom	0	1-het	1	0	0
1q41	0	0	0	0	0	0	0	0	0	0	0	0	0	0	0	0	0	0	0	0	0	0	0	0	0	0
4q22.3	1-hom	1	1	0	1	1-het	1-het	1-het	1-het	1-hom	1-hom	1-hom	1-het	1-hom	1-hom	1-hom	1	1	1-hom	1	1-hom	1-hom	1	1-hom	1	1-het
5q12.3	0	0	0	0	0	0	0	1-het	0	0	0	0	0	0	0	1-het	0	0	0	0	0	0	0	1-het	0	1-het
5q14.1	0	0	0	0	0	0	0	0	0	0	0	0	0	0	0	0	0	0	0	0	0	0	0	0	0	0
6p21.32	1	0	0	1	0	1	0	0	0	1	0	0	1	0	0	1	0	1	1	1	0	0	1	1	0	1
6q26	1-hom	1-hom	1-het	1-het	1-het	1-hom	1-hom	1-het	1-het	1	1-het	0[b]	1-hom	1 - hom	1-het	1	1	1 - het	1-hom	1	1-het	1-hom	1-het	1-het	1-het	1-het
9q34.11	1-het	1-het	1	1-het	1-het	1	1	1	1	1	1	1	1	1	1	1-het	1	1	1	1-het	1	1	1	1	1	1
11q12.2	0	0	0	0	0	0	0	0	0	0	0	0	0	0	0	0	0	0	0	0	0	0	0	0	0	0
12q12	0	1-het	0	0	0	1-het	0	1-het	0	0	0	0	0	0	1-het	0	1	0	1-het	1-het	1-hom	0	0	0	0	0
12q24.31	0	0	1-het	1-het	0	0	1	0	0	1-het	0	0	1	0	0	1-hom	1	0	1	0	0	0	0	1	0	0
13q31.3	0	0	0	0	0	1-het	0	0	0	1-het	0	0	0	0	0	0	1	1-hom	0	0	0	0	0	0	0	0
15q22.2	1-hom	1	1-het	0	0	1-het	1-hom	0	1-hom	1	0	1-hom	0	1-hom	1-hom	1-hom	1	1	1	1	1	1	1	1	1	1
19p12	0	0	0	0	0	0	0	1-het	0	0	0	0	1-het	0	0	0	0	0	0	0	0	0	0	0	0	0
19q12	1	1	1	0	1-hom	0	0	1-het	1-het	0	1-het	1-het	1-het	1-hom	0	1 - het	0	0	1-hom	1-het	0	0	1-hom	0	0	0
20p12.1	0	0	0	0	0	0	0	0	0	0	0	0	0	0	0	0	0	0	0	0	0	0	0	0	0	0

[a] Coverage is low in this region but we do not find any pre-integration sites, suggesting that – despite the rarity of this allele – this individual might be homozygous.
[b] The evidence for the absence of the integration in this patient is presented in Fig. S4.
